# Conservation genetics of the spotted bat: Insights from ancient, historical, and modern samples

**DOI:** 10.1371/journal.pone.0358330

**Published:** 2026-10-07

**Authors:** Faith M. Walker, Daniel E. Sanchez, Michael G. Campana, Robert C. Fleischer, Jeffrey T. Foster, Colin J. Sobek, Cori L. Lausen, Carol L. Chambers

**Affiliations:** 1 School of Forestry, Northern Arizona University, Flagstaff, Arizona, United States of America; 2 Pathogen and Microbiome Institute, Northern Arizona University, Flagstaff, Arizona, United States of America; 3 Center for Conservation Genomics, Smithsonian National Zoo and Conservation Biology Institute, Washington, District of Columbia, United States of America; 4 Wildlife Conservation Society Canada, Kaslo, British Columbia, Canada; UNAM: Universidad Nacional Autonoma de Mexico, MEXICO

## Abstract

Spotted bats (*Euderma maculatum*) are cryptic (nocturnal, volant, and roost solitarily) and thus their biology remains largely undescribed. Only 35 specimens were known to science before the mid-1960s. Hence, they are ideal candidates for genetic studies to uncover population genetic diversity and structure, and thereby inform conservation. We analyzed nuclear microsatellite loci (N = 17) and mitochondrial control region sequences from 121 individuals spanning ancient (up to 2,100 years BP), historical (1904–1991), and contemporary (1994–2025) timeframes across the species’ range in western North America, from Canada to Mexico. Ancient specimens shared haplotypes with modern individuals from the same locality, suggesting long-term population continuity. Microsatellite data revealed isolation-by-distance and a weak genetic cline from the genetically diverse U.S. Southwest core population into the less diverse northern range, consistent with male-biased dispersal and post-Pleistocene range expansion. Mitochondrial data showed stronger regional structure, with distinct haplogroups in the north, central, and southern populations. At a rare cave aggregation, analyses of fine‑scale relatedness identified father–son pairs and a male‑biased sex ratio, suggesting swarming behavior. Our integrative approach across temporal scales highlights the conservation importance of preserving genetically diverse core populations, vulnerable leading-edge populations, and isolated relict lineages.

## Introduction

Population genetics has become one of the pillars of conservation biology since its inception over 50 years ago [1–3]; molecular methods are now powerful and often essential tools for wildlife conservation and management [4–6]. Genetic methods are applied to determine the role of genetic diversity in population viability [7–10] and uncover movement patterns and population structure [11–13]. In a meta-analysis, Shaw et al. [10] found an overwhelming global decline in intraspecific genetic diversity across taxa. They point out that gaining insight into genetic diversity and population differentiation can guide management strategies and conservation decisions, which commonly focus on preserving dispersal and promoting genetic diversity by the protection of migratory corridors [10]. Such insight can also allow identification of management units or different conservation planning for distinct populations [14–16]. Rare or cryptic wildlife in particular can benefit from genetic approaches since they are problematic to study using conventional methods [17].

Many bat species are cryptic because they are nocturnal, small-bodied, and volant, making it problematic to investigate population processes. Because of this, bats are one of the most data deficient groups [18] even though globally they comprise one-fifth of mammalian diversity, with ~1,500 species [19]. In North America there is a pressing need to discern bat population changes in light of threats such as climate change [20–22], white-nose syndrome [23], wind energy [24], persecution [25], and habitat loss [25–27]. Fifty-three percent of North America’s 153 bat species are predicted to have moderate to very high risk of extinction by the year 2039, and 90% of these species are thought to have suffered population declines in the past 15 years [25].

Previous studies have employed molecular tools to determine genetic diversity and population structure to understand and promote the viability of North American bat populations [25,28,29]. For instance, from analyses of genetic structure and inferred dispersal it was possible to understand and forecast the spread of white-nose syndrome in little brown bats (*Myotis lucifugus*) [25]. High genetic diversity and low genetic structure in the proposed endangered tri-colored bat (*Perimyotis subflavus*) was found prior to the arrival of white-nose syndrome [28]. Analyses of genetic structure revealed two matrilineal lineages in the lesser long-nosed bat (*Leptonycteris yerbabuenae*), which helped support designation of two conservation units for the species [29].

With its large pink ears, unique pelage (three white spots on black fur), and echolocation calls that can be heard by unaided human ears, the spotted bat (*Euderma maculatum*) is a charismatic species. However, it is rarely encountered. Only 35 individuals were known to science prior to 1965 [30], and there are currently just 95 specimens in museum collections [31]. In 1899, C. Hart Merriam wrote that “it was among the rarest mammals in the world” [32]. Spotted bats roost solitarily, contributing to their cryptic nature and data-deficiency. The species is patchily distributed in semi-arid regions across western North America from central Mexico to southern British Columbia, Canada. Spotted bats roost in crevices of cliffs that are near (< 10 km) perennial water sources [33] and can traverse various elevations (−53 m to 3,230 m) and vegetation types (xeric lowlands to alpine meadows) while foraging [30]. At the northernmost extent of its range, in British Columbia, the species inhabits semi-arid river valleys characterized by sagebrush, bunchgrass, stands of conifers, and nearby cliff faces [34]. Best [35] found geographic variation in 10 of 16 morphological characters, determining that central Mexico bats were larger in body size than those in other regions. Although robust estimates of population size are unknown, it is presumed that there are between 2,500 and 100,000 spotted bats [36]. It is a Species of Concern in the United States, with different states having varying designations [36,37]. In Canada, fewer than 1,000 adults are thought to occur; although they met the criterion of Threatened D1 (very small population or restricted distribution), they instead received the lesser designation of Special Concern due to a possibility of gene flow from neighboring populations in the U.S. [38].

Patterns of mtDNA from 34 individuals and ecological niche modeling indicated that Mexico constitutes the ancestral range of spotted bats during the Pleistocene, and that Holocene warming resulted in the species’ expansion into its current distribution [39], consistent with evidence from the fossil record [40]. The central Mexico lineage (from the state of Querétaro) appeared to have been isolated due to contraction of climatically suitable conditions during the Holocene, whereas the central and northern regions maintained suitable climatic conditions and underwent population expansion [39]. The central area of the species’ current distribution – Arizona, Utah, and New Mexico – appears to be the spotted bat’s core range, with plentiful cliff habitat and mtDNA haplotypic diversity [33,39]. In contrast, British Columbia, Washington, and Oregon may have been colonized relatively recently by spotted bats, as suggested by the monophyly of mitochondrial data from these populations and the recent emergence of a relatively small area of climatically suitable habitat [39].

Further knowledge of population structure and genetic diversity across the species’ range will promote conservation and management actions for spotted bats. In particular, understanding the dynamics at the leading edge of a species’ range is critical, as peripheral populations often harbor unique evolutionary and ecological characteristics [41]. Rear-edge populations may represent long-term refugia with high genetic distinctiveness and historical continuity, contributing disproportionately to species-level genetic diversity and evolutionary potential [41]. Conversely, leading-edge populations may exhibit reduced genetic diversity as a result of recent colonization, founder effects, and other demographic processes associated with range expansion, yet they play a vital role in future species persistence under climate change by facilitating range expansion [42,43].

Population genetic structure emerges from the cumulative effects of individual movement, dispersal, and reproductive behavior [44,45]. While mitochondrial and nuclear markers are commonly used to infer gene flow and historical connectivity at regional scales, dense sampling at a single locality can additionally reveal fine‑scale genetic processes—such as kinship, sex‑biased dispersal, and patterns of site use—that underlie those broader patterns [46,47]. Nuclear microsatellite markers retain sufficient resolution to infer close familial relationships, allowing relatedness to be examined alongside population structure using the same dataset [6]. Ancient spotted bat remains, dated to between 2,100–10,500 years old, co-occur with extant individuals in a large cave in northern Arizona near Grand Canyon National Park [40], providing a rare opportunity to investigate long-term changes in genetic diversity and population structure.

To investigate the genetic structure and dispersal dynamics across the spotted bat’s range, we conducted a geographically extensive and temporally integrative study using nuclear microsatellite and mitochondrial DNA markers. Genetic data were obtained from contemporary specimens, historic museum collections, and ancient remains, including subfossil bones and mummified specimens. In addition, we assessed kinship and sex-biased dispersal within the natural aggregation of spotted bats from the Grand Canyon area cave, linking fine-scale genetic processes to broader patterns of population structure and historical connectivity across the species’ distribution.

Specifically, we tested the following hypotheses: (i) Contemporary gene flow is predominantly directed from the central core range toward leading-edge populations, consistent with expectations under a scenario of post-Pleistocene range expansion; (ii) Spotted bats from the central core range of the U.S. Southwest exhibit the highest levels of genetic diversity in both mitochondrial and nuclear markers, reflecting long-term persistence through climatic oscillations and the retention of deeply divergent genetic lineages; (iii) Northern populations from the U.S. Northwest and southern British Columbia exhibit reduced genetic diversity and weak genetic structure, consistent with recent colonization during range expansion; and, (iv) Kinship analysis based on nuclear markers reveal close familial relationships and potential sex-biased dispersal within the cave aggregation, linking fine‑scale relatedness patterns to broader patterns of gene flow and dispersal across the species’ range. By combining complementary perspectives on evolutionary history and contemporary population dynamics through mitochondrial DNA and nuclear microsatellite markers across ancient, historical, and contemporary samples, we provide a comprehensive view of genetic structure and diversity in spotted bats, enabling the investigation of long-term population continuity, range expansion, kinship, and conservation-relevant patterns across spatial and temporal scales.

## Materials and methods

### Genetic sample collection

We obtained genetic samples from 121 spotted bats (Fig 1; S1 Table), including contemporary field and museum specimens collected between 1994 and 2025, historical museum specimens collected between 1904 and 1991, and ancient remains recovered from a northern Arizona cave and radiocarbon dated to 120–2,100 years before present (BP). Radiocarbon dating was conducted by Beta Analytic (Miami, FL, USA). Spotted bats were captured over stock ponds and at the cave entrance using mist-nets during summers from 2009 to 2013. Field samples consisted of 2-mm wing punches or buccal swabs [17] (Whatman OmniSwab, Whatman International Ltd., Maidstone, UK) and were stored at −80 °C until DNA extraction. Historical samples (skin clips, skeletal muscle, or organs) from 65 spotted bats were obtained from 15 museums (S1 Table).

**Fig 1:**
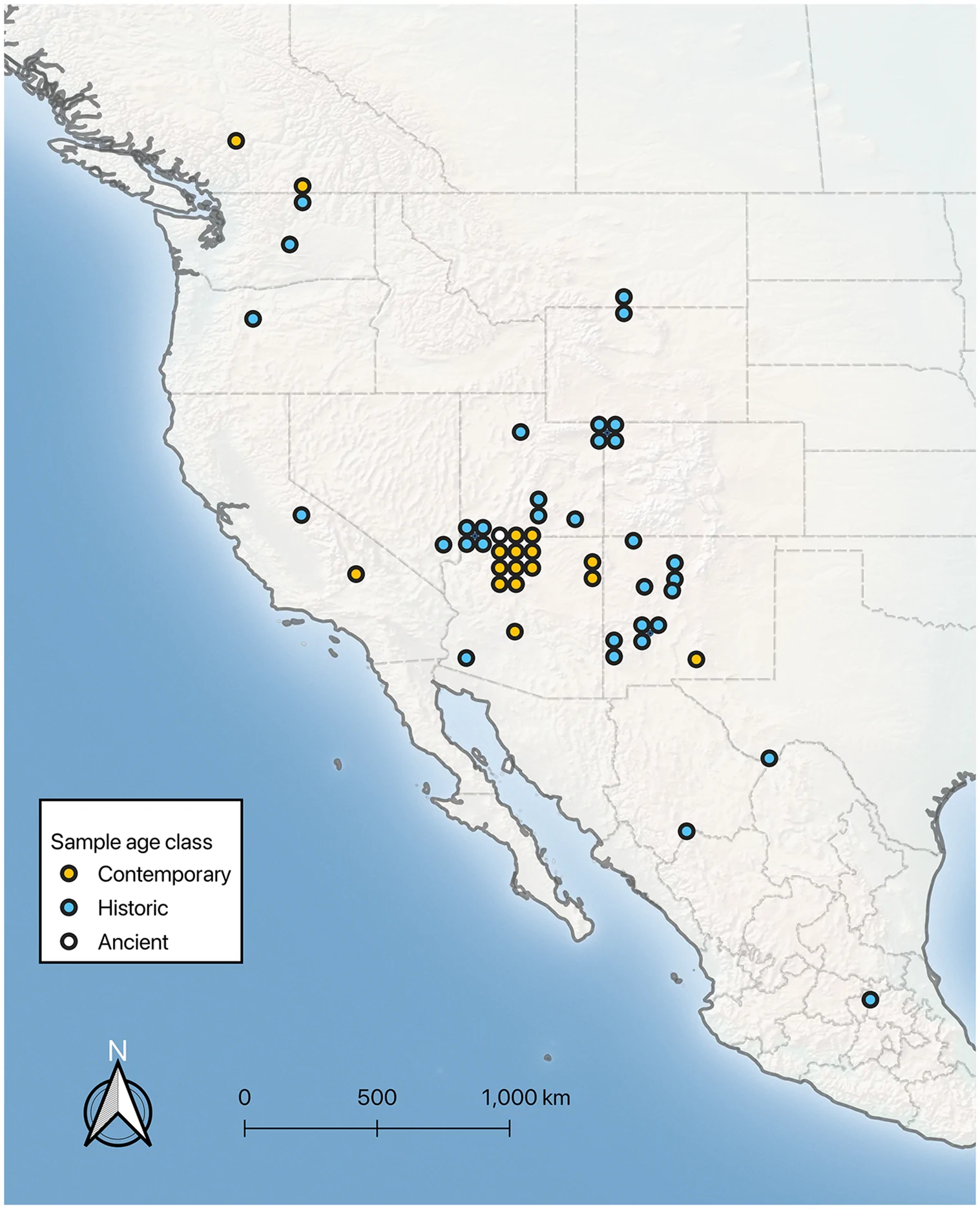
Distribution of contemporary, historic, and ancient spotted bat sampling localities. Points are displaced to distinguish the different temporal classification where sampling coordinates overlap.

### DNA extraction, microsatellite genotyping, and mtDNA sequencing

We extracted DNA from contemporary samples using a Qiagen Blood and Tissue Kit (Qiagen, Valencia, CA, USA). For historical and ancient samples we extracted DNA using the phenol:chloroform procedures of Fleischer et al. [48]. DNA extractions of ancient samples were performed in two dedicated ancient DNA labs (NAU and the Smithsonian) that adhere to international standards that minimize risk of contamination [49].

We PCR-amplified the 10 microsatellite loci of Walker et al. [31] on MJ Research PTC-200 thermal cyclers in 12 µL reactions. For spotted bats captured at the cave, we PCR-amplified all 17 loci [31] (except for five samples, for which we amplified 10). Historical and ancient samples were amplified at least twice. Reactions contained 1X PCR Rxn Buffer (Invitrogen), 2 mM MgCl_2_ (Invitrogen), 0.2 mM dNTPs, 0.5 U/µL Platinum Taq DNA polymerase (Invitrogen), 0.05 µg/µL Ultrapure non-acetylated Bovine Serum Albumin (Ambion), 0.5 µM forward primer, 0.5 µM reverse primer, molecular grade H_2_O, and 2 µL DNA template (at 2 nm/µL). Thermal cycling conditions were as follows: 8 min denaturation at 94 °C; 35 cycles of 94 °C for 30 s, T_a_ 54 or 61 °C [31] for 30 s, and 72 °C for 1 min; 72 °C extension for 10 min. We performed fragment analysis on an Applied Biosystems 3130 Genetic Analyzer and visualized results with GeneMapper 4.0 software.

We designed primers to target 196 bp of the 1,200 bp mitochondrial control region sequenced in Boston et al. [50] (forward: Dloop_196 bp, GATGCTTGGACTCAACACTG; reverse: RevL16517_600, GTCCTGTAACCATTAAGTTCAC). PCRs contained 1X PCR Rxn Buffer (Invitrogen), 2 mM MgCl2 (Invitrogen), 0.2 mM dNTPs, 1 U/µL Platinum Taq DNA polymerase (Invitrogen), 0.02 µg/µL Ultrapure non-acetylated Bovine Serum Albumin (Ambion), 0.5 µM forward primer, 0.5 µM reverse primer, molecular grade H_2_O, and 2 µL DNA template (at 20 ng/µL) in a 10 µL reaction. Thermal cycling conditions were as follows: 3 min denaturation at 95 °C; 35 cycles of 95 °C for 30 s, T_a_ 58 °C for 30 s, and 72 °C for 1 min; 72 °C extension for 10 min. We diluted the PCR product 1:10 before sequencing on an Applied Biosystems 3130 Genetic Analyzer using BigDye 3.1 Terminator Chemistry. We used Sequencher 5.0 to assemble forward and reverse sequences for all samples, verify or correct base calls, and to generate a consensus sequence.

### Microsatellite data analysis

#### Locus behavior and genetic diversity.

We re-amplified samples with equivocal results and private alleles, and inherently problematic samples were subjected to increased thermal cycles (to 45). We used Micro-Checker 2.2.3 [51] to evaluate presence of null alleles (with 1,000 randomizations). For an indication of the power of discrimination of the final panel of loci, we calculated P_*ID*_ [52]. We used GENEPOP v4.2 (http://genepop.curtin.edu.au/) to examine expected (H_E_) and observed (H_O_) heterozygosity, departures at each locus from Hardy-Weinberg equilibrium, and linkage disequilibrium between each locus pair. Because allelic diversity is sensitive to sample size, we performed rarefaction using the program HP-Rare 1.0 [53]. We employed 2-sample t-tests to compare heterozygosity and rarified allelic richness between the northern (Canada and U.S. Northwest) and central (U.S. Southwest) regions. We did not include the southern region (central Mexico; Querétaro) in these tests because sample size was low (N = 2).

#### Genetic structure.

For microsatellite data, we investigated the genetic differentiation among populations designated *a priori* [39]. Populations consisted of north (U.S. Northwest; British Columbia, Canada; N = 12) and central (U.S. Southwest and Rocky Mountains; N = 101) individuals. AMOVA was performed in Arlequin v3.5.2.2 [54], which employs nonparametric permutation methods (20,000 iterations) to test for significance of the variance components of the within and among-population levels of genetic structure. We also tested whether there was a difference in genetic structure between historical (1931–1991; n = 37) and modern individuals (1994–2014; n = 64). To evaluate the magnitude of genetic structure, we calculated Weir and Cockerham’s [55] estimator of Wright’s *F*-statistic pairwise among the two *a priori* population groupings in the R v4.5.2 package *hierfstat* v0.5-11 [56]. We calculated 95% confidence intervals (1000 bootstrap iterations) to evaluate significance and uncertainty for the derived differentiation statistics. Following recommended guidelines [57], we used a discriminate analysis of principal components (DAPC) in R package *adegenet* v2.1.11 [59,60] to graphically summarize the degree of differentiation among the *a priori* population assignments and predict membership probabilities for individuals within those groupings. DAPC is a multivariate method designed to detect clusters of genetically similar individuals by enhancing between-cluster differentiation while reducing within-cluster variation [58]. In running the DAPC, we selected the number of principal component axes using the recommended K-1 retention approach [59,60], where K was the number of *a priori* populations (K = 2), and the final number of PC axes retained for the discriminate analysis was 1.

To examine movement patterns between central and northern populations, we tested recent migration with BAYESASS v3.0.5.6 [61]. This program uses a Bayesian method to estimate relative migration rates. We optimized mixing parameters (dM = 0.3, dA = 0.4, and dF = 0.3) and ran three times with different starting seeds for 21 million iterations, with 1,000 intervals between samples. Consistent results were indicated by all independent runs converging and having concordant posterior probability distributions in Tracer v1.7 [62]. We discarded the first 60% of the MCMC iterations as burn-in and calculated 95% highest posterior density intervals from the MCMC data using R package *bayestestR* v0.17.0 [63]. We repeated this analysis with a standardized dataset by randomly subsampling the central population so that sample sizes were the same for both populations.

We also investigated *de novo* population structure across the range. We performed a Principal Coordinates Analysis (PCoA) in GenAlEx v6.51b2 [64] and used ggplot2 for visualization [65]. We used the Bayesian clustering program STRUCTURE v2.3.4 [66] together with Structure Harvester v0.6.94 [67]. We assessed the most likely number of genetic clusters (K) using the methods of Evanno et al. [68] and the Ln P(D) as determined in CLUMPAK [69]. We used an admixture model with correlated allele frequencies, 5 runs per K (K = 1–6), a burn-in of 50,000, and 200,000 Markov chain Monte Carlo (MCMC) iterations, and withheld sampling location information. We then conducted a spatial principal component analysis (sPCA) in *adegenet* v2.1.11 [59,60]. sPCA is a multivariate method to detect cryptic spatial structure and uses Moran’s index to assess spatial autocorrelation among groups or individuals. A positive spatial autocorrelation suggests that neighboring individuals are genetically similar (global structure), while a negative spatial autocorrelation points to genetic dissimilarity among neighbors (local structure) [70]. We down-sampled the dataset due to the AZ cave site having disproportionate representation (39 individuals with shared geographic coordinates) such that we randomly sampled 3 males and 3 females from that locality. This resulted in a dataset of 80 individuals. We jittered location data (factor = 0.03 for latitude and longitude) due to multiple individuals tending to be recaptured in the same geographic coordinates (e.g., mist netting) and used the Gabriel graph method as a starting network. We applied global.rtest and local.rtest [70] functions to test in *adegenet* for local and global structure using 999 permutations under the null hypothesis of no spatial structure. Given these results, we retained the first PCA axis, reflecting global structure. We used R package *interp* v1.1-6 [71] to interpolate 200 lagged principal scores across the geographic extent of the observed latitude and longitude values. To support the sPCA, we used GenAlEx v6.51b2 [64] to examine spatial autocorrelation of genetic and geographic distance over all individuals and by sex.

#### Genetic relatedness and sex bias within the cave.

We estimated mean pairwise genetic relatedness from multilocus genotypes using the program kinship v1.2 [72]. We tested for a sex difference in mean pairwise relatedness between sex classes by performing randomization tests (10,000 permutations via Resampling Stats v5.0.2 [73]). We identified the most likely mother and/or father of each individual using the log-likelihood-based program CERVUS v2.0 [74]. The mean probability of excluding a randomly selected unrelated bat from parentage was > 0.999. Because there was no prior knowledge of maternity, we examined each individual (candidate offspring) with all females (candidate mothers) and all males (candidate fathers), in separate simulations of 10,000 cycles. We set parameters at 50% of candidate parents sampled, 95% of loci typed, and an error rate of 0.3% (the upper error rate quantified for the present protocol). We assigned parentage when all of the following criteria were met: (i) Likelihood-Odds Ratio (LOD) > 3.0 (Marshall *et al*. 1998); (ii) delta > 95% [74]; (iii) a maximum of one allele mismatched by no more than two base pairs; and (iv) the mismatch was associated with scoring issues.

Interpreting sex bias requires understanding whether observed patterns are species‑specific or reflect broader ecological processes associated with cave use. Many bat species use caves seasonally for different functions, including maternity roosting, day roosting, or swarming behavior associated with mating and dispersal [75,76]. We examined sex ratios across all nine bat species captured at the cave from July 2007 to April 2016 to provide comparative context (Fisher’s exact test). This allowed us to distinguish whether a sex-bias observed in spotted bats was unusual or consistent with patterns expected at caves used primarily for swarming rather than maternity. Such context is essential for interpreting kinship patterns and for understanding how cave use may influence dispersal and gene flow in spotted bats.

### mtDNA data analysis

We aligned 116 sequences of the spotted bat control region using R package *DECIPHER* v3.2.0 [77] to evaluate structure and diversity. We obtained haplotypes and generated a median-joining network [78] (ε = 0) of those haplotypes using R package *pegas* v1.3 [79] to infer genealogical groupings at the state/province level. We then calculated haplotype and nucleotide diversity summaries both globally and for *a priori* populations. We calculated the number of polymorphisms (segregating sites; S), parsimony informative sites (PIS), number of haplotypes (Hap), haplotype diversity (Hd), and nucleotide diversity (π) using R packages *ape* v5.8-1 [80], *phyloch* v1.5-5 [81], and *pegas* [79]. All specimens were used for global diversity summaries, but we excluded two specimens from the population groupings: one from Wyoming (MSB:Mamm:114512) and the other from Colorado (MSB:Mamm:22756). Using the same marker as the current study, it was previously shown that haplotypes for this pair of specimens form a distinct grouping within the northern Rocky Mountains, USA, sharing a well-supported common ancestor with the northwestern haplogroup containing Oregon, Washington, and British Columbia [39]. In that study, this relationship was consistent among phylogenetic trees inferred with different optimality criteria. However, considerable divergence between the northwestern haplogroup and the Rocky Mountain haplogroup suggests their pooling into a single population would confound insight that could be gleaned from Oregon, Washington, and British Columbia alone. Hence, we removed the Rocky Mountain pair [WY (n = 1), CO (n = 1)] from consideration. Using the same global and population groupings we also evaluated their differentiation in R package *hierfstat* [56] using global and pairwise Fst [55] with significance calculated using 95% confidence intervals from bootstrapping (1000 iterations).

### Ethics statement and permits

All necessary permits were obtained for the described study, which complied with all relevant regulations*.* Bats were captured and handled using guidelines of the American Society of Mammalogists [82] and with the approval of Northern Arizona University’s Institutional Animal Care and Use Committee (permit numbers 07–006-R2, 14–008, and 15–006), Arizona Game and Fish Department (SP706855), the Navajo Nation Fish and Wildlife Department (Special Permit 908), and the British Columbia Government (permits to C.L.L. MRCB20–598305, MRCB15–163558). Captured bats were held for 30 minutes or less and were released after collection of genetic samples. No bats suffered injury or mortality in this study. We withheld the cave name and location due to conservation concerns and the potential risk of disturbance. All bat handlers were vaccinated against rabies and used appropriate personal protective equipment.

### Inclusivity in global research

Additional information regarding the ethical, cultural, and scientific considerations specific to inclusivity in global research is included in the Supporting Information (S1 Checklist).

## Results

### Genotyping and sequencing success

From the cave we collected six deceased spotted bats, which were up to 2,100 years old (conventional radiocarbon age = 2100 + /- 40 BP, 1450 + /- 40 BP, 300 + /- 30 BP, 180 + /- 30 BP, 124.9 + /- 0.5 BP, and 120 + /- 40 BP). We captured 69 spotted bats; 55 of which were from northern Arizona, of which 39 were from the cave. For 37 bats we took a wing punch, and for the remainder we collected DNA via buccal swabs. For museum samples, 46 yielded multilocus genotypes over 10 microsatellite loci (Loci: EUMA8, EUMA6, EUMA2, EUMA12, EUMA55, EUMA18, EUMA36, EUMA47, EUMA32, EUMA39). We thus genotyped a total of 115 individual spotted bats for 10 loci (Fig 1; S2 Table), and the subset of 39 contemporary individuals from the cave for 17 loci (S3 Table). All loci adhered to Hardy-Weinberg expectations after correction for multiple tests, and none exhibited evidence of linkage disequilibrium or null alleles, as was found during marker development and testing [31]. Only 4.7 x 10^-16^ in a million pairs of spotted bats selected at random would be expected to share a multilocus genotype. For the mitochondrial control region, 116 contemporary and historic individuals and all six ancient specimens were successfully sequenced (see S4 Table for Genbank accession numbers).

### Genetic diversity

For microsatellite markers, mean observed and expected heterozygosity were both 0.71, and mean allelic richness was 7.3 (S5 Table; Table 1). The number of alleles per locus ranged from 5–12, with a mean of 7.3 (S5 Table). Spotted bats from the central region had significantly greater allelic richness (rarified) (*P* = 0.03) and expected heterozygosity (*P* = 0.0019) than those from the northern region (Table 1). The northern region had no private alleles, and the central region had 28 (Table 1). For mtDNA, we recovered 31 control region haplotypes among the 116 individuals with 40 segregating sites and 35 parsimony informative sites (Table 1). The central population (26 haplotypes; 101 individuals) carried the highest diversity estimates, where haplotype diversity was ~ 2.5 × greater than the northern population (2 haplotypes; 11 individuals) and greater by an order of magnitude in nucleotide diversity. The southern population had only two individuals represented by a single haplotype. Therefore, the remaining diversity metrics were zero among the two specimens originating from Querétaro, Mexico.

**Table 1:** mtDNA and microsatellite marker diversity summaries by spotted bat population and for all individuals. Parameters for mtDNA include number of individuals (Nseq), segregating sites (S), parsimony informative sites (PIS), number of haplotypes (Hap), haplotype diversity (Hd), and nucleotide diversity (π). Parameters for microsatellites include mean number of individuals genotyped at each locus, observed heterozygosity (HO), expected heterozygosity (HE), rarified allelic richness, and private alleles. For microsatellites, South (Querétaro, Central Mexico) was not included due to small sample size (N = 2).

mtDNA							Microsatellite				
Population	N_Seq_	S	PIS	Hap	Hd	π	Mean # individuals per locus	H_O_	H_E_	Rarified allelic richness	Private alleles
North	11	1	1	2	0.327	0.002	12	0.630	0.584	3.38	0
Central	101	24	16	26	0.844	0.012	87	0.715	0.692	3.86	28
South	2	0	0	1	0	0	–	–	–	–	0
All individuals	116	40	35	31	0.876	0.025	101	0.71	0.71	–	–

North: Washington, Oregon, British Columbia.

Central: Arizona, New Mexico, Utah, Nevada, Texas, California, Colorado (n = 3), Montana, CHIHUAHUA.

South: QUERÉTARO

All individuals: QUERÉTARO, CHIHUAHUA, Arizona, New Mexico, Utah, Nevada, Texas, California, Colorado, Wyoming, Montana, Oregon, Washington, British Columbia

Excludes Rocky Mountain haplogroup (Wyoming (n = 1), Colorado (n = 1)).

### Genetic structure: Mitochondrial data

The median-joining network (Fig 2) allowed us to identify at least five regionally distinctive haplogroups across the range of spotted bats, each exhibiting 5–16 mutational steps (n ≥ 2 individuals) to another haplogroup. This still included a distinct northwestern (Oregon, Washington, British Columbia), Rocky Mountain (Wyoming, Colorado), southwestern (Arizona, New Mexico, Colorado, Utah, Nevada, Chihuahua), and southern (Querétaro) haplogroup at the southernmost portion of the species range. However, a California haplogroup became more apparent with an additional sample collected from the region in 2021. Texas appeared to exhibit a sixth regional haplogroup, but with a single individual collected in 1968 it remains unclear whether this haplotype is extant or exclusive to the region. The southwestern haplogroup was characterized by extensive haplotypic richness, genealogical uncertainty (e.g., alternative links and median vectors), and range coverage—extending from Chihuahua, Mexico to northern Colorado/Utah (~1500 km), and in longitude from Nevada/southwestern Arizona to eastern New Mexico (~830 km). The most predominant haplotype (n = 34 individuals) of this region produced a starlike pattern and gave the greatest geographic diversity—largely throughout the four corners region (Arizona, New Mexico, and Utah) but extending nearly the entire latitude of Arizona. Notably, this predominant haplotype was shared by 5/6 of the ancient specimens (≤ ~ 2000 years BP) and 14/38 contemporaneous individuals at the same cave locality. The remaining ancient specimen only differed by one SNP and shared a haplotype with two contemporaneous individuals of the same cave locality, as well as two individuals captured in Canyon de Chelly, Arizona, in 2014. The broader structure patterns were supported with elevated and significant F_ST_ (global = 0.86) values at 0.839 [0.706–0.914, 95% CI] between central and north populations, 0.898 [0.822–1.000, 95% CI] between central and south populations, and 0.987 [0.950–1.000, 95% CI] between north and south populations.

**Fig 2:**
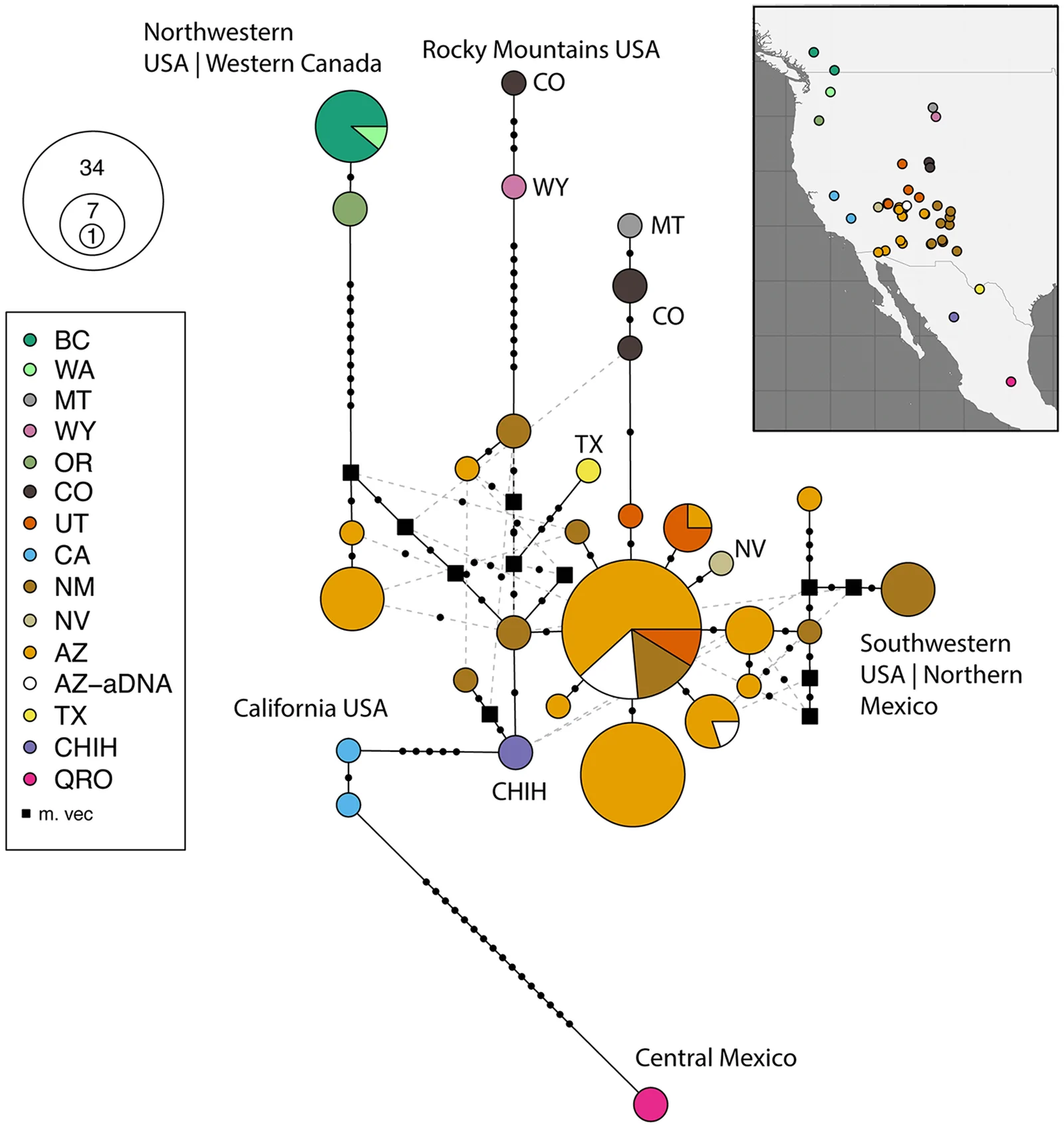
Median-joining haplotype network for 116 spotted bat individuals using a 193 bp fragment of the mitochondrial control region. Pies and slices (haplotypes) are given color fill corresponding to 14 states/provinces/municipalities that individuals had originated from and sized relative to the number (n) of specimens of a haplotype (transformed as $\frac{n}{\pi }$ for simplified sizing). Square-shaped nodes indicate median vectors, dashed lines indicate alternative links, and small circles indicate the number of SNPs differing between two nodes. An inset map is provided in the top right of the plot for geographic reference of sampling points and their region categories. Key for state/province/regional municipality are found in S1 Table.

### Genetic structure: Microsatellite data

In the AMOVAs, the proportion of the variance in allele frequencies among populations (north, central) was significant (12%, *P* = 0.001) (S5 Table). AMOVA did not differ by specimen age (1%, *P* = 0.36), nor did H_E_ (paired t-tests: t(9) = 0.63, *P* = 0.50). *A priori* analyses of structure showed low but discernible genetic differentiation among north and central regions, as evidenced by the significance of Weir and Cockerham’s F_ST_ [55] (F_ST_ = 0.061, [0.019–0.128, 95% CI]). The sexes did not differ in F_ST_ (F_ST_ = 0.03, *P* = 0.97). A DAPC scatter plot with overlapping 95% ellipses indicated weak differentiation among pre-assigned population groupings and that individual membership probabilities (Q) were largely assigned individuals to the central population (Fig 3). The PCoA, which does not use pre-assigned population groupings, appeared similar to the DAPC plot (S1 Fig). sPCA scree plots and eigenvalues (Fig 4A-4B) indicated significant global (*P* = 0.04) and local (*P* = 0.02) structure, with a perceptible gradient of differentiation from central to north and some observable spatial structure within the southwestern U.S. (Fig 4D-4E). The BAYESASS analysis suggested higher migration from the central population into the northern population (British Columbia, Washington, Oregon) than in the reverse direction (Fig 5). However, this pattern was not recovered when the central population was subsampled to match the size of the northern population, with migration estimates then indicating no detectable recent movement between populations. This sensitivity to sample size indicates that estimates of directional migration are not robust and should be interpreted cautiously. *De novo* analyses on the STRUCTURE results indicated that the most likely number of populations was one. However, program STRUCTURE is sensitive to skewed population sizes. Spatial autocorrelation analysis showed isolation by distance, as evidenced by bats in close proximity being significantly more related and those at the greatest distance being significantly less related; there was no difference between same-sex pairs, as indicated by overlapping bootstrap error bars (S2 Fig).

**Fig 3:**
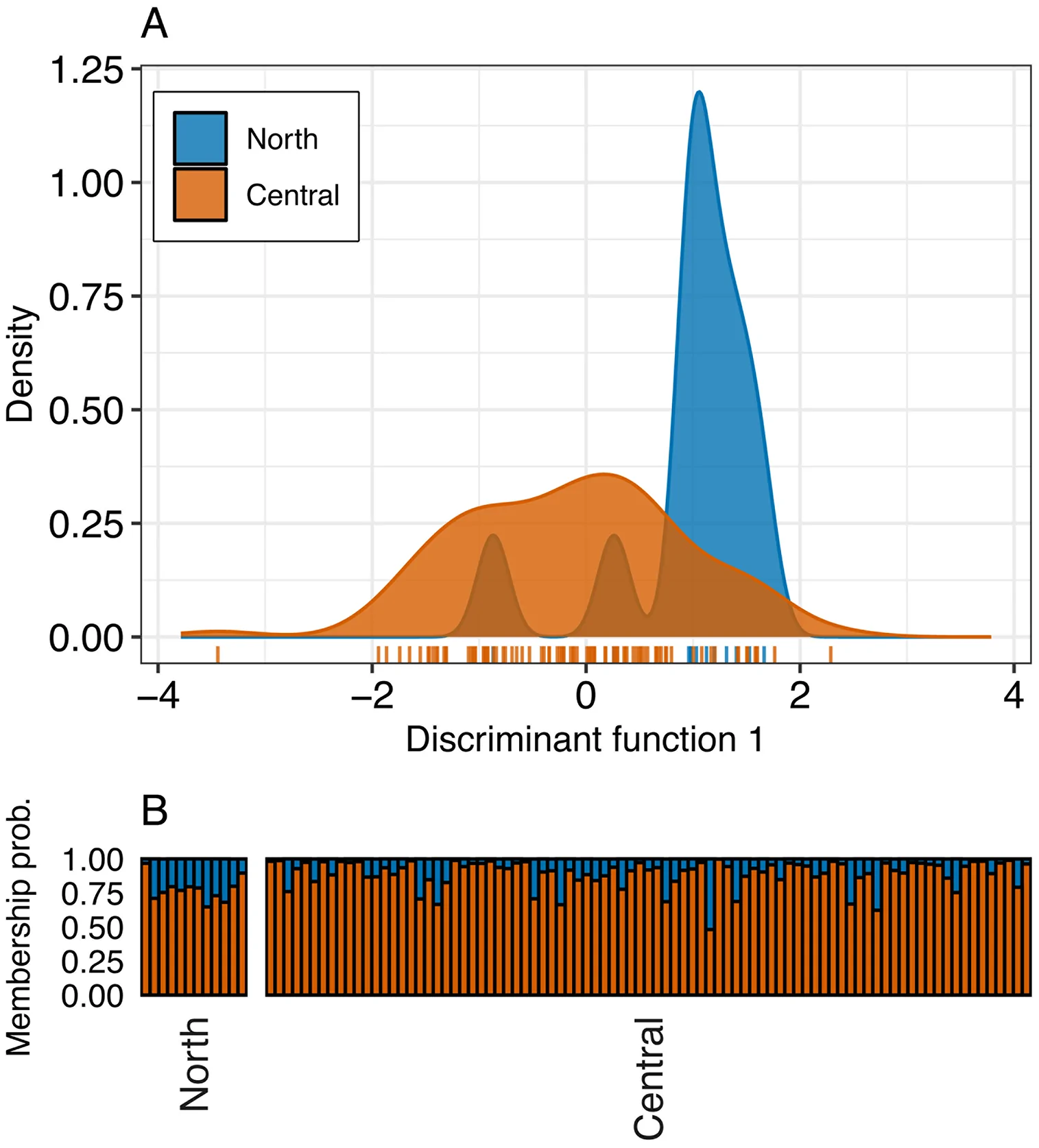
Discriminate analysis of principal components (DAPC) for two *a priori* population groupings of spotted bat microsatellite data indicate weak genetic differentiation between populations. A) Density plot showing distribution of individual discriminant scores along a single biologically informative discriminant axis (K – 1 PC axes retained); B) Q plot with each bar representing an individual with color fill showing membership probability to one of two *a priori* populations.

**Fig 4:**
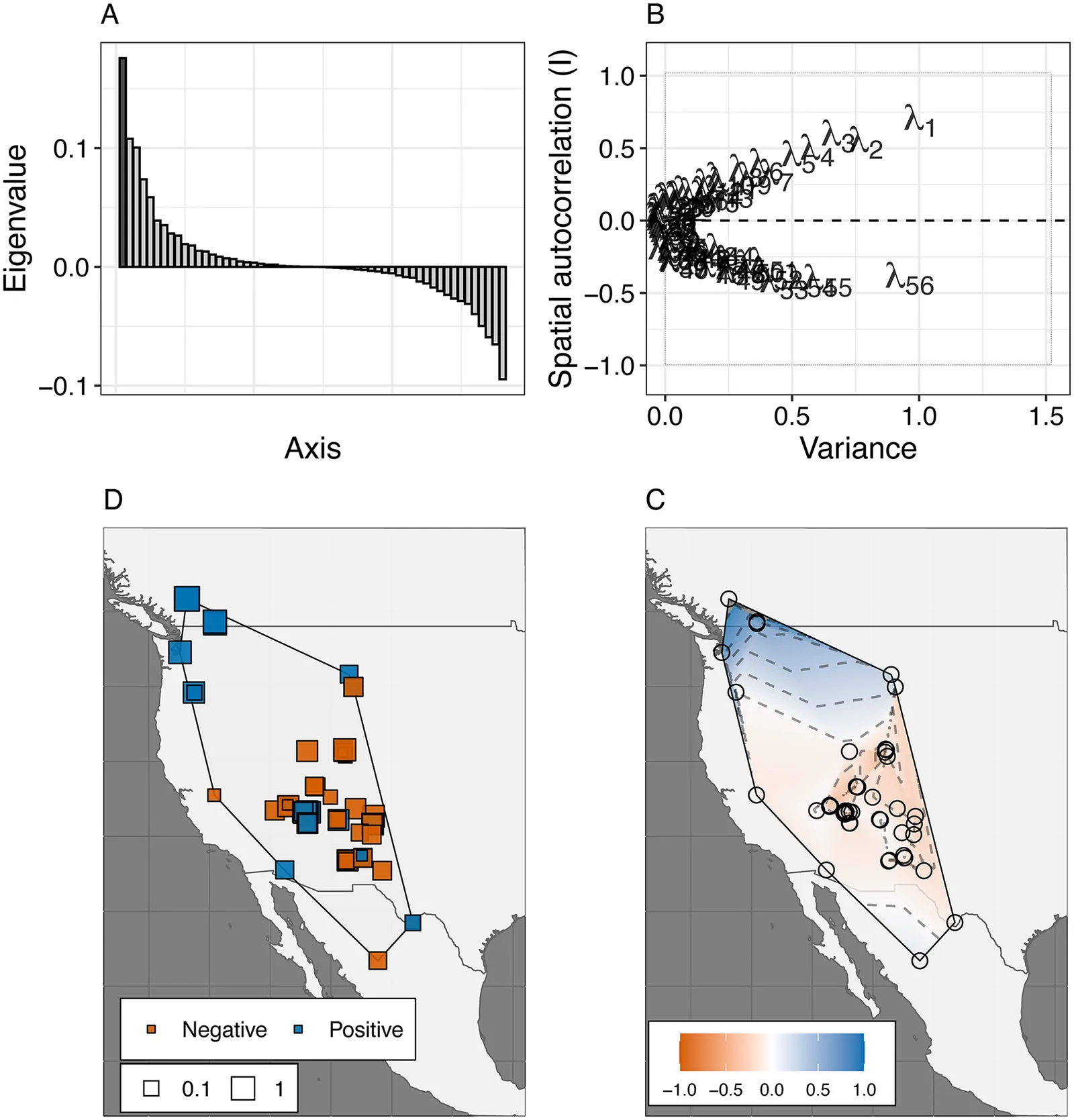
Spatial principal component analysis (sPCA) results for spotted bat microsatellite data. A) eigenvalues, with positive bars reflecting global structure (*P* = 0.04) and negative bars reflecting local structure (*P* = 0.02); the darkened bar indicates the PC axis retained for the analysis; B) screeplot for both positive and negative values on Moran’s I, also showing the difference in variance between lambda 1 (first PCA – global structure) and lambda 58 (last PCA – local structure), suggesting a cline in genetic variation; C) different colored lagged scores with box size representing the magnitude (larger boxes are more differentiated and smaller boxes are less differentiated than their neighbors). Positive and negative values signal differentiation; and D) spatial interpolation of the lagged scores indicate subtle south to north clines in genetic structure. The interpolation plot includes a convex polygon denoting the spatial extent of the analysis, open (jittered) points to reference observed data, and contour lines (dashed).

**Fig 5:**
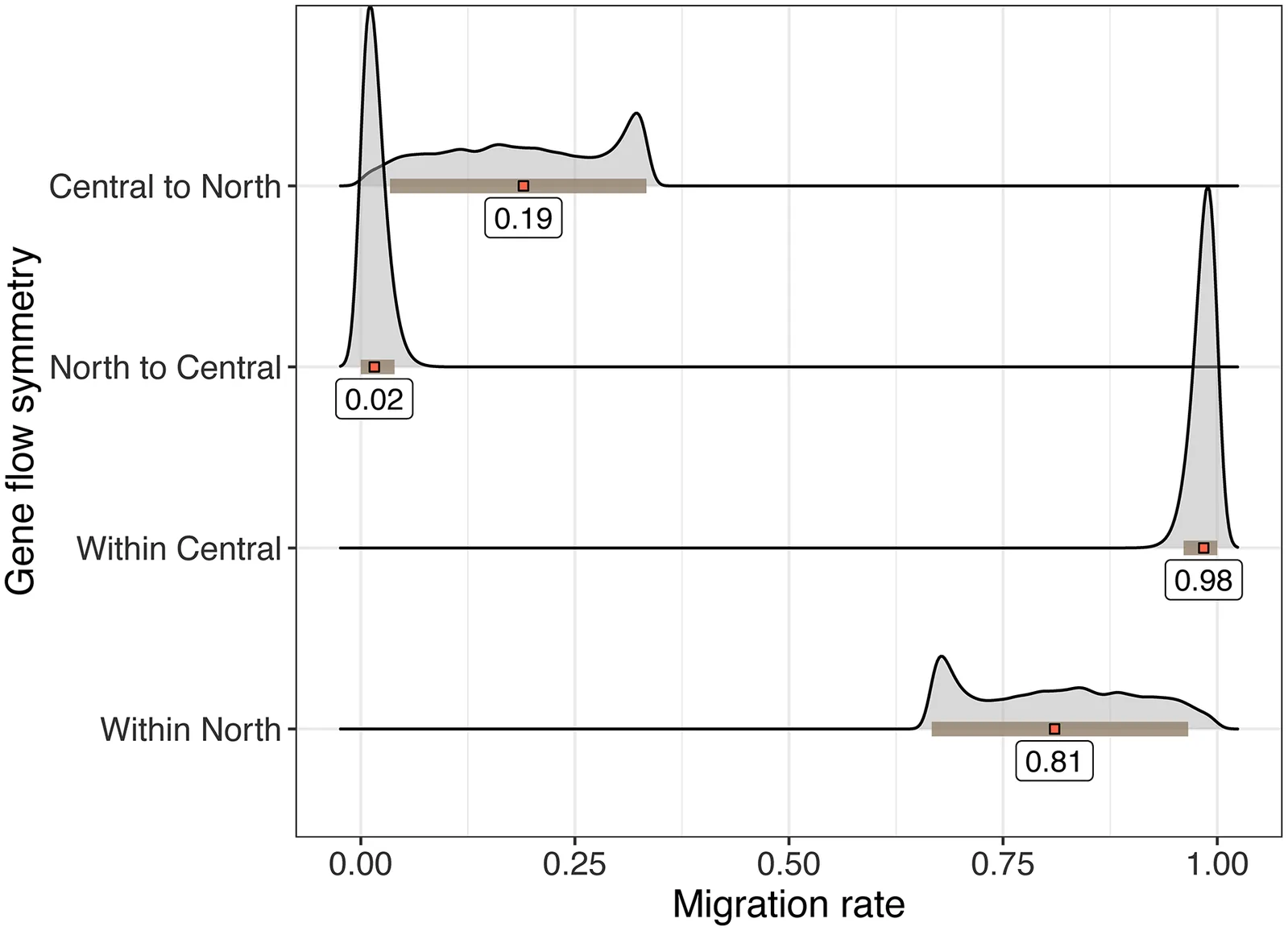
Results of BAYESASS analysis for northern (BC, OR, and WA) and central (U.S. Southwest) spotted bats. Initial analyses suggested higher migration from the central population into the northern population than in the reverse direction; however, this pattern was not recovered when the central population was subsampled to match the size of the northern population, indicating sensitivity of migration estimates to sample size. Plots show the posterior density of the migration rate, defined as the proportion of individuals in the recipient population assigned as migrants from the donor population. Bars represent 95% highest posterior density intervals, and squares indicate posterior means.

### Genetic relatedness within the cave and sex bias

The delta criterion [74] was 4.94 at the 95% confidence level and 2.00 at the 80% confidence level. Two male pairs met the parent-offspring criteria. All four individuals were captured in 2009, the most productive sampling year. There were no other genetically compatible bats, and both accepted father-sons shared an allele at all loci. Additionally, we detected two pairs of close male relatives, and one pair of close male-female relatives.

Among spotted bats that used the cave, there was a sex bias toward males (31 males; 8 females). Of the nine bat species that were captured, six had strong biases toward males (S7 Table), and the sex-bias was significant for all species except *Parastrellus hesperus* (Fisher’s exact test; P = 0.006 after Bonferroni correction). Spotted bats and other species with a strong male sex-bias had the highest number of male captures in April and May (S7 Table).

## Discussion

Spotted bats have a central core population in the U.S. Southwest that is genetically diverse and weakly differentiated from the northern population in the U.S. Northwest and southern British Columbia, Canada, consistent with expectations for a leading-edge range shaped by post-Pleistocene expansion [42]. Estimates of recent migration were sensitive to sampling design and did not provide robust support for directional asymmetry. However, genetic patterns from both mitochondrial and nuclear markers are consistent with a northward cline in gene flow and reduced genetic diversity in northern populations. These observations are consistent with a history of post-Pleistocene range expansion; however, the relative contributions of founder events, population bottlenecks, migration, and isolation by distance cannot be distinguished from the present data.

Together, these patterns support ongoing connectivity between regions, rather than isolation. It is plausible that gene flow from the genetically diverse core contributes to the persistence of northern populations, although the direction and magnitude of recent migration remain uncertain. The weak differentiation observed in both F-statistics and DAPC further reinforces this interpretation of connectivity. Encouragingly, the updated haplotype network reveals even greater diversity in the southwestern U.S., supporting a scenario of both Holocene expansion and strong dispersal capabilities. The star-like pattern of the haplotype network in the central region further supports demographic expansion, likely driven by warming during the Holocene. This region, centered around Utah, New Mexico, and Arizona (where the 10,500-year-old specimen was found), contains the most frequent haplotype, shared by both ancient and modern individuals. Increasing climatic suitability likely enabled bats to exploit the abundant cliffs of the Colorado Plateau and Intermountain West. In contrast, the leading-edge populations in the U.S. Northwest and southern British Columbia show signs of recent colonization, supported by the presence of only two haplotypes across that region (although it is possible that more locations could reveal additional haplotypes). Notably, California consistently forms its own haplogroup, which may reflect geographic or ecological isolation—possibly due to the Sierra Nevada range acting as a barrier. These findings underscore the conservation importance of both core and peripheral populations: while the northern range may be less exposed to climate extremes, its lower diversity and recent colonization make it vulnerable; conversely, the central region may serve as a reservoir of genetic variation critical for future adaptation.

Interpretation of genetic structure in spotted bats must consider marker choice and sampling design. The mitochondrial control‑region fragment analyzed here is necessarily short due to constraints imposed by historical and ancient samples and, as a maternally inherited marker, reflects only female lineage history. As such, mtDNA captures deeper phylogeographic structure but cannot fully represent contemporary gene flow or sex‑biased dispersal and is therefore interpreted alongside nuclear microsatellite data that better reflect recent population dynamics. Modern sampling was also uneven, with many individuals originating from a single cave that represents a rare aggregation site. This dense local sampling was intentional to enable fine‑scale kinship analyses but could influence population‑level inference if uncorrected. Accordingly, we also down‑sampled cave individuals for clustering and spatial analyses and interpreted these results cautiously, while mitochondrial analyses, AMOVAs, and broad‑scale diversity comparisons remain robust to this sampling structure. Together, these considerations emphasize that our conclusions are strongest when integrating multiple marker types across spatial scales.

We did not find genetic structure across time, likely due to the long generation time of bats or to population size stability. The sharing of a haplotype between the 2,100-year-old spotted bats and their contemporaries at the cave suggests population stability in the central region over this time period. The stronger signal of genetic structure in mtDNA than nuclear DNA is likely a reflection of gene flow taking place via male movements, although it may also reflect lower effective population size of haplotypic variants or higher mutation rate of microsatellites. Male-biased dispersal is thought to be common in bats (e.g., [83]). Given this, it is interesting that two father-son pairs were found in the cave. Chambers et al. [33] captured multiple male spotted bats at the cave in the early 2000’s and suggested that they roost singly since the cave is so large. The general male bias across species suggests swarming behavior [75,76], which refers to the aggregation of individuals—typically at caves or mines—where bats from multiple colonies repeatedly circle, chase, and land at these sites, engaging in social interactions that serve purposes such as mating, orientation of young to hibernation sites, and preparation for hibernation. In little brown bats (*Myotis lucifugus*), groups were more likely to be male, and males preferred to associate with males that they grouped with for more than one night [75]. Swarming in this case was thought to be juvenile males learning site locations and adult males cooperating for enhanced mating opportunities [75]. Additionally, for the species with a male-bias in our study, it may be that the cool conditions in the cave (mean 8°C ± SD 0.19, mean humidity 51% ± 0.55 [84]) do not provide a sufficiently warm microclimate for maternity sites. Female spotted bats roosted on south-facing cliffs while males roosted randomly, suggesting selection for a temperature range that might be warmer [33].

The central Mexico haplotype (from the state of Querétaro) differed from other haplotypes by a high number of substitutions per site, even compared to northern Mexico. Ecological niche modeling suggested that beginning 130,000 years ago the central Mexico population started to become isolated due to contraction of suitable climate [39]. Additional investigation of spotted bats in this region is hampered by the paucity of museum specimens, captures, and acoustic data. Only two specimens were available for this study, and there has only been one detection in this area in over 40 years. This bat was found in 2021 in the state of Hildalgo, 130 km south of Querétaro, which is the southernmost record of the species [85,86]. Sanchez et al. [39] found a notable absence of suitable climate space that extends into central Mexico and that it is patchy in Chihuahua, Mexico, and Big Bend, Texas. They suggested that the genetic and geographic isolation, difference in morphology [35], and significance of this lineage to the species’ evolutionary legacy justify designation of an evolutionary significant unit (ESU) [87,88] deserving of special conservation attention [39].

In addition to phylogeographic divergence, spotted bats in the central core area differ behaviorally from those in the north (reviewed in [39]). Bats in Arizona traveled long distances to forage (77 km roundtrip, straight line distance) [33] and had 300 km^2^ home ranges [33,89]. In the Fraser and Okanagan Valleys, British Columbia, spotted bats roosted 6–10 km from where they foraged [90,91] and home ranges were less than 5 km^2^ [92]. The significantly greater travel by bats of Arizona and likely other areas of the U.S. Southwest may be due to lower prey or water availability, or greater interspecific competition than in the north [93]. Sanchez et al. [39] hypothesized that spotted bats responded to lower habitat quality brought on by drought or high temperatures by dispersal-promoting behavior, suggesting that their long travel distances were due to lower quality habitat of drier semi-desert areas. Indeed, spotted bats were found at new foraging sites in New Mexico during a drought [94].

Although spotted bats may have an enhanced capacity for dispersal in changing climates and their diet of diverse noctuid, geometrid, and lasiocampid moths may promote their wide geographic distribution [95], they are subjected to an increasing array of anthropogenic disturbances [21]. For instance, in the U.S. Southwest, climate change and overgrazing may reduce water availability and decrease vegetation key to recruiting moths and occur at a rate exceeding bat response. It is unclear how spotted bats in the north will react to climate change. One scenario is that increasing frequency of wildfires will convert forests to dry grasslands at lower elevations. This could increase suitable habitat for spotted bats where there are crevices or caves in cliffs and nearby water [39]. Hence, we encourage conservation measures for the leading-edge population(s) as they may become important to future distribution of the species. It is also important to minimize local extirpations in the core range so that genetic diversity has the potential to spread to the north.

We suggest that the reduced genetic diversity, small population size, and different behavior of the northern population(s) warrants designation as an ESU. The central population of spotted bats can be considered a Conservation Unit due to its differentiation from the other populations and larger size. There may be discrete populations adjoining this region. We suggest that a genomic approach (e.g., [96]) with additional samples from northern California, northern Nevada, Idaho, Montana, Wyoming, Colorado, and Mexico (Durango, Coahuila) will help resolve genetic structure between the core area and neighboring areas, and Mexico. Together, these results highlight how integrating ancient, historical, and modern genetic data can illuminate evolutionary processes and inform conservation strategies for cryptic species, underscoring the importance of preserving genetically diverse core populations, vulnerable leading-edge populations, and potential isolated trailing populations that may represent relict lineages critical to the species’ evolutionary legacy.

## Supporting information

S1 Fig Principal coordinates analysis of spotted bat microsatellite data with 95% ellipses (multivariate t-distribution). (DOCX)

S2 Fig Spatial genetic structure for a) pairwise spotted bats, and b) same-sex pairs across their distribution. The observed relationship (blue) is significant where it lies outside the dotted lines (red) denoting the 97.5% and 2.5% confidence intervals for each distance class and where the error bars do not overlap with 0.
(DOCX)

S1 Table Collection data for all spotted bat (*Euderma maculatum*) samples used in this study. (XLSX)

S2 Table Multilocus genotypes for 10 microsatellite markers across 115 spotted bat individuals. (XLSX)

S3 Table Multilocus genotypes (17 loci) for 39 contemporary spotted bats from the cave in northern Arizona. 0 indicates no data obtained.
(XLSX)

S4 Table Mitochondrial control region data for the spotted bat, *Euderma maculatum*, specimens. (XLSX)

S5 Table Summary AMOVA table for spotted bats from northern and central regions. (XLSX)

S6 Table Observed and expected heterozygosity, and allelic richness of the 10 microsatellite loci used in this study. (XLSX)

S7 Table Bat captures at the cave in Northern Arizona from 2007 to 2016. A) Sex-bias across species; and, B) Species with the strongest male sex-bias, by month.
(XLSX)

S1 Checklist Inclusivity in global research. (DOCX)
