# Supplementary material for: Conservation genetics of the spotted bat: Insights from ancient, historical, and modern samples
Source: PLoS One. 2026 Oct 7;21(10):e0358330. doi: 10.1371/journal.pone.0358330 (PMC13645066; doi:10.1371/journal.pone.0358330)
Supplement: Supplementary file 1 [file pone.0358330.s001.docx]

**S1_Fig.** Principal Coordinates Analysis of spotted bat microsatellite data with 95% ellipses (multivariate t-distribution).
