# Supplementary material for: Conservation genetics of the spotted bat: Insights from ancient, historical, and modern samples
Source: PLoS One. 2026 Oct 7;21(10):e0358330. doi: 10.1371/journal.pone.0358330 (PMC13645066; doi:10.1371/journal.pone.0358330)
Supplement: Supplementary file 2 [file pone.0358330.s002.docx]

**S2_Fig**. Spatial genetic structure for a) pairwise spotted bats, and b) same-sex pairs across their distribution. The observed relationship (blue) is significant where it lies outside the dotted lines (red) denoting the 97.5% and 2.5% confidence intervals for each distance class and where the error bars do not overlap with 0.

a)

r

| Distance (km) | r | U | L |
| --- | --- | --- | --- |
| 0 | 0.0110609 | 0.00526435 | -0.00432023 |
| 150 | -0.00714274 | 0.00578027 | -0.00649218 |
| 500 | 0.00369084 | 0.00652795 | -0.00699781 |
| 1000 | -0.0111024 | 0.00487994 | -0.005623 |

b)

R values by Population

r

| Distance | r | U | L |
| --- | --- | --- | --- |
| Female | -0.00486841 | 0.00721073 | -0.00549225 |
| Male | -0.00242493 | 0.00373923 | -0.00280629 |
