# Supplementary material for: Conservation genetics of the spotted bat: Insights from ancient, historical, and modern samples
Source: PLoS One. 2026 Oct 7;21(10):e0358330. doi: 10.1371/journal.pone.0358330 (PMC13645066; doi:10.1371/journal.pone.0358330)
Supplement: Supplementary file 3 [file pone.0358330.s010.docx]

Inclusivity in global research

PLOS’ policy on inclusivity in global research aims to improve transparency in the reporting of research performed outside of researchers’ own country or community and ensures that PLOS publications reporting global research adhere to high standards for research ethics and authorship. Authors of relevant research articles may be asked to complete the questionnaire below, which outlines ethical, cultural, and scientific considerations specific to inclusivity in global research. This questionnaire may be requested when researchers have travelled to a different country to conduct research, if research uses samples collected in another country, research with Indigenous populations or their lands, or if research is on cultural artefacts. Researchers travelling to another country solely to use laboratory equipment will not normally be required to complete the questionnaire. However, the questionnaire can be requested at the journal’s discretion for any submission – if you have been requested to complete this questionnaire by the PLOS journal you submitted to, please do so.

Please complete the questionnaire below and include this as a Supporting Information file with your manuscript. Note that if your paper is accepted for publication, this checklist will be published with your article in the supporting information files. Please ensure that you reference the checklist in the main body of your manuscript. We suggest adding a subsection ‘Inclusivity in global research’ to your Methods section and adding the following sentence: “Additional information regarding the ethical, cultural, and scientific considerations specific to inclusivity in global research is included in the Supporting Information (SX Checklist)”

The questions have been designed to be applicable to a wide range of study types, and there are subsections for both human subjects research and non-human subjects research. If any of the questions are not relevant to your research please mark them as “N/A” as appropriate.

**Ethical considerations, permits and authorship**

*This section is applicable to all research types.*

Provide details as to who granted permissions and/or consent for the study to take place in the Methods section of your manuscript. This should include the names of **all** ethics boards, governmental organizations, community leaders or other bodies that provided approval for the study. If individuals provided approval refer to these people by their role or title but do not list their name(s).

Reported on page number: 13

If there were any deviations from the study protocol after approval was obtained please provide details of these changes in the Methods section of your manuscript.
Did this study involve local collaborators that are residents of the country where the research was conducted or members of the community studied? If you do not have any authors from said communities, please provide an explanation for this below.

Reported on page number: N/A

The study did not include local collaborators as co‑authors. The authors have an established history of collaboration with Navajo Nation scientists, including prior co‑authorship with a Navajo Nation zoologist on earlier spotted bat research (Chambers et al. 2011*). For the present study, we invited participation from the same individual and other staff within the Navajo Nation Fish and Wildlife Department; however, no local collaborators were available to participate due to time and capacity constraints.

The study was conducted under authorization from the Navajo Nation Fish and Wildlife Department, and all research activities were carried out in accordance with the approved permit.

*Chambers, C. L., M. J. Herder, K. Yasuda, D. G. Mikesic, S. M. Dewhurst, W. M. Masters, and D. Vleck. 2011. Roosts and home ranges of spotted bats (*Euderma maculatum*) in northern Arizona. Canadian Journal of Zoology 89(12):1256-1267.

Everyone listed as an author should meet PLOS’ criteria for authorship and all individuals who meet these criteria should be included in the author byline, rather than the acknowledgements. For further information please see the journal’s Authorship Policy.

**Human subjects research (e.g. health research, medical research, cross-cultural psychology)**

Did you obtain written informed consent from a representative of the local community or region before the research took place? How did you establish who speaks for the community? Details of written informed consent obtained from study participants should be reported separately in the Methods section of your manuscript.

N/A

How did members of the local community provide input on the aims of the research investigation, its methodology, and its anticipated outcome(s)?

N/A

When engaging with the local community, how did you ensure that the informed consent documents and other materials could be understood by local stakeholders?

N/A

Will the findings of the research be made available in an understandable format to stakeholders in the community where the study was conducted (e.g. via a presentation, summary report, copies of publications, etc.)? Please provide details of how this will be achieved.

N/A

**Non-human subjects research using specimens/ animals collected as part of the study, or those housed in archival collections. Examples include archaeology, paleontology, botany and zoology.**

Did the permission you obtained from a local authority to perform the study include an agreement on access to outputs and benefit sharing? This may include procedures to enable fair distribution of the benefits and resources arising from the research performed. Please include any details of Prior Informed Consent and Benefit Sharing Agreements obtained. These may be required by field-specific regulations, for example the Convention on Biological Diversity (CBD) and the associated Nagoya Protocol.

The research was conducted under a scientific research permit issued by the Navajo Nation Fish and Wildlife Department, which authorized access to the study area and permission to carry out the described field activities. No separate agreement regarding access to research outputs or formal benefit‑sharing arrangements was required or established beyond the terms of this permit.

Prior Informed Consent was obtained through the Navajo Nation Fish and Wildlife Department as part of the permitting process. The permit governed the scope of activities, ethical conduct of research, and compliance with Navajo Nation regulations. No additional Access and Benefit Sharing (ABS) agreement under the Convention on Biological Diversity or Nagoya Protocol was applicable to this study. No genetic resources were accessed or transferred under an ABS framework, and all work was conducted in accordance with applicable tribal, state, and federal regulations.

If the material used in your study was imported, please A) provide the year it was imported and B) indicate whether permits were obtained to import/export the materials used, C) provide details of any permits obtained. If this information is not available, please indicate this.

The material in the study was not imported.

If you used archival specimens, please state how the material used in your study was acquired by the institute it is held in and provide details of any permits obtained for the original excavations/ sample collection. If this information is not available, please indicate this.

The archival material was obtained from 15 museums. The details of how the specimens were acquired or the permits they obtained to do so is not available.

How was the potential cultural significance of the materials collected in your study to local communities considered in your research design? Were Indigenous peoples and/or local researchers and institutions involved with archaeological excavations / collection of specimens? If so, please provide a description of their involvement.

The potential cultural significance of research activities was addressed through the Navajo Nation Fish and Wildlife Department’s permitting and oversight process, which ensures that wildlife research conducted on Navajo Nation lands is consistent with cultural, ethical, and regulatory considerations. The study involved biological sampling and did not include archaeological excavation, handling of cultural materials, or disturbance of culturally sensitive locations.

Prior Informed Consent was obtained via a scientific research permit issued by the Navajo Nation Fish and Wildlife Department. Although the authors have collaborated with Navajo Nation researchers on prior projects, no Indigenous collaborators or local institutional staff participated directly in this study due to their time and availability constraints. No additional community consultation or collaborative agreements were required for the approved scope of work.

If your manuscript includes photographs of human remains please indicate whether authors obtained permission from descendants or affiliated cultural communities to do so.

N/A
